# Strabismus and Strabismus Surgery in the U.S. Veterans Health Administration: Foundational Analyses of Electronic Health Record Data from 2000 to 2022

**DOI:** 10.3390/jpm15020040

**Published:** 2025-01-21

**Authors:** John H. Lillvis, Michael Feehan, Treefa Shwani, Amy E. Millen, Gregory E. Wilding, Karen M. Allison, Leah A. Owen, Margaret M. DeAngelis

**Affiliations:** 1 Department of Ophthalmology, Ross Eye Institute, Jacobs School of Medicine and Biomedical Sciences, State University of New York, University at Buffalo, Buffalo, NY 14203, USA; jhlillvi@buffalo.edu (J.H.L.); mfeehan2@buffalo.edu (M.F.); treefash@buffalo.edu (T.S.); leah.owen@cchmc.org (L.A.O.); 2 Veterans Administration Western New York Healthcare System, Buffalo, NY 14212, USA; 3 M/A/R/C Research, Irving, TX 75038, USA; 4 Neuroscience Program, Jacobs School of Medicine and Biomedical Sciences, State University of New York, University at Buffalo, Buffalo, NY 14203, USA; 5 Department of Epidemiology and Environment Health, School of Public Health and Health Professions, State University of New York, University at Buffalo, Buffalo, NY 14214, USA; aemillen@buffalo.edu; 6 Department of Biostatistics, School of Public Health and Health Professions, State University of New York, University at Buffalo, Buffalo, NY 14214, USA; gwilding@buffalo.edu; 7 Department of Ophthalmology, Flaum Eye Institute, University of Rochester, Rochester, NY 14642, USA; karen_allison@urmc.rochester.edu; 8 Division of Ophthalmology, Department of Surgery, Cincinnati Children’s Hospital Medical Center, Cincinnati, OH 45229, USA; 9 Department of Ophthalmology and Visual Sciences, University of Utah School of Medicine, The University of Utah, Salt Lake City, UT 84132, USA; 10 Department of Population Health Sciences, University of Utah School of Medicine, The University of Utah, Salt Lake City, UT 84132, USA; 11 Department of Obstetrics and Gynecology, University of Utah School of Medicine, The University of Utah, Salt Lake City, UT 84132, USA; 12 Department of Biochemistry, Jacobs School of Medicine and Biomedical Sciences, State University of New York, University at Buffalo, Buffalo, NY 14203, USA; 13 Genetics, Genomics and Bioinformatics Graduate Program, Jacobs School of Medicine and Biomedical Sciences, State University of New York, University at Buffalo, Buffalo, NY 14203, USA

**Keywords:** electronic health records, strabismus, strabismus surgery, prevalence and incidence, Veteran, Veterans Health Administration, VINCI

## Abstract

**Background/Objectives:** Strabismus, or eye misalignment, has not been well-described in U.S. military Veterans. This study was undertaken to characterize Veterans with a strabismus diagnosis as well as those who underwent strabismus surgery. **Methods:** A retrospective analysis of electronic health records (EHR) from the Veterans Health Administration (VHA) was conducted using patient data from 2000 to 2022. VHA-enrolled Veterans ≥ 18 years with strabismus-related International Classification of Diseases (ICD) codes and/or Current Procedural Terminology (CPT) codes were identified. Total and demographic (age group, sex, race, and ethnicity) stratified prevalence and incidence rates were calculated, as well as sex-stratified residual lifetime risk. **Results:** A total of 321,639 patients had a strabismus diagnosis, with most (320,107) identified by ICD code (CPT code only = 1532). The peak prevalence was 2.29% in the 2022 VHA fiscal year (1 October 2021 to 30 September 2022) with a median annual age-adjusted incidence rate of 168.9/100 000 enrollees. Age-adjusted lifetime risk was 10.19% for males and 11.03% for females. Significant differences by age group, sex, race, and ethnicity were identified for strabismus prevalence (*p* < 0.001), strabismus diagnosis types (*p* < 0.001), and between patients with strabismus who either did or did not have surgery (sex *p* < 0.05, all others *p* < 0.001). Compared with other U.S. adult populations, VHA Veterans have similar or higher prevalence, annual incidence rates, and lifetime risk of a strabismus diagnosis, with demographic factors significantly affecting the rates and types of strabismus. Notably, despite lower prevalence and incidence than other racial groups, a higher percentage of African American patients with strabismus underwent surgery, contrasting with published Medicare data. Inconsistencies between ICD and CPT codes highlight potential miscoding and/or missing codes, with reliance on ICD code diagnoses potentially underestimating strabismus prevalence. **Conclusions:** Further characterization of factors affecting strabismus risk among these patients may help improve strabismus diagnosis and management for many US Veterans. This foundational study serves as a platform for detailed predictive analyses in determining risk outcomes for individuals. This includes better identification of at-risk individuals, informing effective resource allocation for treatment.

## 1. Introduction

Strabismus, the constant or intermittent loss of binocular alignment, is a heterogenous disorder in adults associated with adverse functional and psychosocial impacts [1]. It may be long-standing, from untreated or decompensated childhood strabismus, or new-onset and secondary to trauma, orbital inflammation, or neurologic causes. Available data on the prevalence and incidence of strabismus among adults in the United States (U.S.) come from several sources, with rates that vary widely across populations and study designs. The highest reported prevalence comes from the 1971–1972 National Health and Nutrition Examination Survey (NHANES), which estimated 6.1% of the population aged 55–75 years to have a heterotropia and 19.7% to have a heterophoria [2]. By directly examining a sample of 10,126 persons for ocular motility, however, this study was designed to identify as many cases as possible, and resulted in estimates higher even than those from similar screening studies in other populations [3,4,5,6,7]. In contrast, published reports of Medicare claims data among Medicare Beneficiaries (≥65 years of age) estimated that only 0.68% of beneficiaries had a billing code for strabismus in a given year [8,9]. With regards to adult strabismus incidence and risk, the Rochester Epidemiology project reported an annual incidence of 0.05% in patients aged 19 years and older in Olmstead County, Minnesota, and a 4% lifetime risk of receiving a diagnosis of adult-onset strabismus [10]. More recently, a study of the Intelligent Research in Sight (IRIS) patient registry reported on the strabismus diagnoses documented by 7200 participating ophthalmologists during the years 2013 to 2016 [11]. Although not reporting on population prevalence, the percentage of patients aged 20 years and older seen by an ophthalmologist with a billing code for strabismus ranged from 1.49% to 2.84% in IRIS registry data.

Despite the U.S. Veterans Healthcare Administration (VHA) being the largest integrated health system in the U.S. and the healthcare provider for approximately half of all U.S. military Veterans, strabismus and other eye diseases in Veterans receiving care through the VHA have not been adequately studied. Most studies of eye disease in Veterans are limited to a single hospital or region rather than nationally, with a small number of exceptions mostly focusing on cataract and cataract surgery [12,13,14,15,16,17,18]. Strabismus in particular has been reported as an outcome in several case series of Veterans with traumatic brain injury (TBI), with a prevalence among those patients ranging from 0.6% (when using billing codes) to >20% (when relying on patient examination) [19,20,21,22]. In the total VHA population, it is possible that the frequency and types of strabismus differ from the civilian population due to an increased prevalence of certain strabismus risk factors such as TBI and stroke among Veterans [23,24,25].

Electronic health record (EHR) data from the VHA offer a particularly rich resource for studying strabismus and eye disease in Veterans. Over 9 million Veterans are enrolled in the VHA, and the system has a single unified EHR, the Computerized Patient Record System (CPRS), which has been implemented at all VHA facilities since 1999. CPRS data may be accessed through the VA Informatics and Computing Infrastructure (VINCI), an initiative developed to increase research access to VHA data. VINCI hosts statistical and informatics tools in a secure work environment and has been previously used to study cataract surgery at VHA facilities as well as COVID-19 and eye disease [12,13,17,26]. Nearly half of all Veterans are enrolled in the VHA, with enrollee demographics similar to non-enrollee demographics [27]. A survey of VHA-enrolled Veterans, however, found that those who utilized VHA care were more likely to identify as African American or female, and reported lower household income and more medical conditions than VHA-enrolled Veterans who were not utilizing VHA services [28]. Although reported to be generally better or equal to non-VHA care, the quality of care in the VHA varies across the VHA system [29]. Access to eye care services may depend upon geographic differences; VHA eye clinics range from community-based clinics staffed by optometrists or comprehensive ophthalmologists to large academic hospitals with larger teams, including trainees (students, residents, or fellows) and subspecialist providers. Thus, for a specific eye condition such as strabismus, a patient may have greater access to a provider specialized in diagnosing and treating their condition if all of their care is located at an academic center with a fellowship-trained specialist. In contrast, a patient receiving care in a rural satellite clinic may require visits at multiple locations (e.g., a primary care provider, comprehensive eye care provider, or subspecialist), possibly requiring travel to another VHA facility or referral outside the VHA. These extra steps and provider variation increase the potential for a missed diagnosis, wrong diagnosis, or delay in treatment.

The aim of the present retrospective study was to identify and characterize patients in the VHA with a strabismus diagnosis and those who underwent strabismus surgery. National-level CPRS data from 2000 to 2022 were queried using VINCI research tools to identify patients with either an International Classification of Diseases (ICD) code diagnosis of strabismus or those who underwent strabismus surgery, as determined by Current Procedural Terminology (CPT) codes. Using national-level VHA enrollment data, the annual prevalence of strabismus, and the incidence rate of new strabismus cases, the lifetime risk of strabismus was estimated for the total VHA population. Enrollment data by sex, race, ethnicity, and patient age were also used to calculate stratified prevalence estimates. Secondary analysis included characterization of the types of strabismus diagnosed in this population and the amount and types of strabismus surgery performed.

## 2. Materials and Methods

The study was conducted according to the guidelines of the Declaration of Helsinki and the protocol was approved by the Veterans Administration Western New York Health Care System Research and Development Board under project number 1632919, approved 6 January 2022. A waiver of consent was granted under the revised common rule Category 4 (iii) for secondary research uses of identifiable private information. Data access was also subject to approval of an application through the national VHA Data Access Request Tracker (DART) system.

This study characterized strabismus diagnoses and strabismus surgery among all adult Veterans aged 18 years and older enrolled in the VHA healthcare system from 2000 to 2022. To identify cases, EHR data were analyzed from the VA Office of Information and Technology’s Corporate Data Warehouse (CDW), the national repository for CPRS data. For this study, the CDW was queried as a Structured Query Language (SQL) database using databasing and statistical analysis tools hosted by VINCI. To protect patient confidentiality, all EHR data, whether containing patient identifiers or not, were stored and analyzed within a secure, remote VINCI work environment.

To identify VHA-enrolled patients with strabismus, the records of adult Veteran patients treated at all VHA facilities during the calendar years 2000–2022 were queried. All Veterans aged 18 years and older with either an ICD code for strabismus or a CPT code specific to strabismus surgery were included. Non-Veterans as well as patients with inconsistent demographic data as described below were excluded. The ICD9 codes for strabismus range from 378.0 to 378.9 and the ICD10 codes range from H49 to H51. Corresponding ICD9 and ICD10 codes were mapped to a single group of strabismus categories as per Repka, et al. [11].

Strabismus surgery was determined by the presence of CPT codes 67311–67345. Since these CPT codes are specific to strabismus, patients found to have a CPT code for strabismus surgery, but no documented ICD code for strabismus, were also considered to be strabismus cases in this study. The date of first diagnosis was determined to be the first occurrence of a strabismus-specific ICD or CPT code. The date of first surgery and date of first diagnosis for each specific strabismus ICD category were also identified.

Demographic data on sex, race, ethnicity, date of birth, and date of death were collected from VA demographic tables, and the age at first strabismus diagnosis was calculated. Race and ethnicity categories were taken as reported in the EHR. Patients with more than one identified race category, except for unknown, were classified as “Multiple Races”. Race and ethnicity data were analyzed separately and not combined into a single variable. A history of any medical condition deemed to be a connected to a Veteran’s military service was determined using the “ServiceConnectedFlag” field in patient demographics tables. Non-Veteran patients receiving care through the VHA system were excluded prior to VINCI data provisioning. Patients with multiple listed sexes (*n* = 7) or multiple documented years of death (*n* = 21) in our patient cohort were identified and excluded. Patients with multiple birthdates were also identified (*n* = 71; all with two birthdates). The two birthdates were then manually examined, and if one birthdate was a clear typographical error, such as a date occurring within the last 15 years, the other birthdate was included as the valid birthdate. If both birthdates were potentially valid, the patient was excluded (*n* = 43).

Data collection in VINCI was performed using RStudio software (version 4.3) [30,31]. In brief, database access tools were used to interface with the CDW SQL database and retrieve tables of EHR data on patient demographics, clinical diagnoses, and surgical details. Tables were then joined and manipulated using the tidyverse and data.table packages in R (version 4.3) [32,33]. Only de-identified, aggregate data were included in study tables and figures. As Veteran patients may utilize multiple VHA facilities across different geographic regions, a unique, universal health identifier that links EHR data was assigned to each patient to merge patient information.

National VHA enrollment data, which were used to establish the population denominator for prevalence and incidence rate calculations, was obtained from the Current Enrollment Cube, a Pyramid Analytics webtool that is part of the VHA Support Service Center Capital Assets (VSSC) and is accessible from the VHA intranet. Enrollment data in the Current Enrollment Cube are reported by Veterans Administration Year (FY) (1 October through 30 September) instead of by calendar year. Tables containing total enrollment, as well as enrollment stratified by sex, race, ethnicity, and age group, were generated for all available years, exported to comma-separated value (.csv) files, and uploaded into the VINCI environment. Life expectancy tables for Veterans, which were available stratified for age group and sex, were also downloaded from the VA [34].

All data analyses were performed using RStudio. Descriptive statistics are reported for the entire time period, 2000–2022. Prevalence and incidence rates were calculated by VA fiscal year due to the availability of enrollment data. Annual cumulative prevalence was calculated by dividing the number of non-deceased patients with a diagnosis of strabismus during or prior to that fiscal year by the fiscal year’s enrollment. Annual incidence rates were calculated as new cases per enrolled patients at risk during each fiscal year. Stratified prevalence and incidence rates were calculated in a similar manner for sex, race, ethnicity, and age-group stratified data. Residual lifetime risk was calculated using age and sex-stratified incidence rates and VA life expectancy tables [34].

Statistical tests were performed using R. Two-sided tests were used with statistical significance defined as a *p* < 0.05. Fisher’s exact test was used within the R package CBCgrps to compare demographics between patients who underwent strabismus surgery and patients with a strabismus diagnosis without surgery [35]. A paired Wilcoxon Signed Rank Test (two groups) or Friedman Test (three or more groups) were used to compare prevalence and incidence rate data stratified by demographic characteristics. A Chi-square test of independence was performed using the base R package to compare the distribution of the types of strabismus diagnoses by demographic characteristics.

## 3. Results

### 3.1. Prevalence, Incidence, and Lifetime Risk of Strabismus

**Figure 1:**
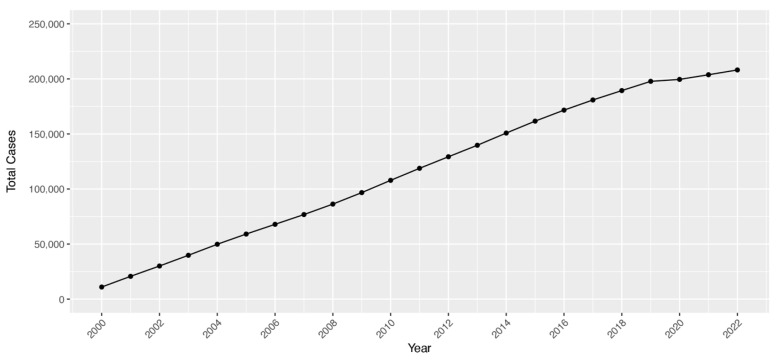
Total strabismus cases among Veterans in the Veterans Health Administration population annually, 2000–2022.

**Figure 2:**
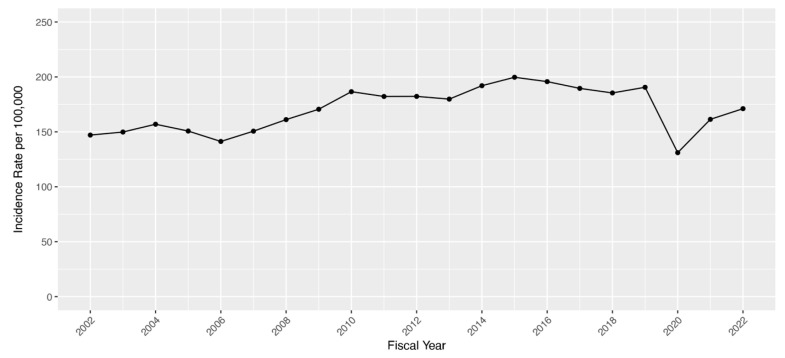
New strabismus cases per 100,000 Veteran enrollees in the Veterans Health Administration, 1 October 2002 to 30 September 2022.

**Figure 3:**
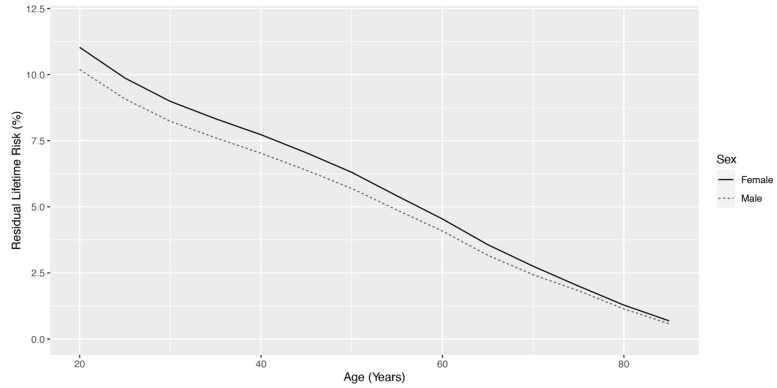
Residual lifetime risk of strabismus diagnosis by age for male and female Veterans enrolled in the Veterans Health Administration.

A total of 321,639 unique Veteran patients were identified as a strabismus case by the presence of either an ICD diagnosis or CPT code for surgery from the VHA EHR period of 1 January 2000 to 31 December 2022. The total number of VHA-enrolled Veterans with current or previous strabismus steadily increased over the study period (Figure 1), and the prevalence increased from 0.41% in FY 2002 to a peak of 2.29% in FY 2022. A median of 14,977 Veterans were identified per year, with a median annual age-adjusted incidence rate of 168.9 strabismus cases per 100,000 enrollees. Incidence rates increased from 2002 until 2015, plateaued, and then showed a sharp decrease in 2020 during the COVID-19 pattern (Figure 2), with new cases by year demonstrating a similar pattern. Using available VA mortality tables stratified by age and sex, the risk of being a strabismus case for Veterans in the VHA was found to be 10.19% for males and 11.03% for females. Residual lifetime risk by age is plotted in Figure 3.

### 3.2. Number and Types of Strabismus Diagnoses

The most common ICD strabismus type in the total VHA population was heterophoria (27.04% of patients with strabismus; 19.91% of strabismus diagnoses), followed by exotropia (24.61% of patients; 18.12% of diagnoses), esotropia (17.49% of patients; 12.88% of diagnoses), and paralytic strabismus (17.32% of patients; 12.75% of diagnoses) (Table 1). The ratio of exotropia (XT) diagnoses to esotropia (ET) diagnoses (XT:ET ratio) was 1.40. The majority of patients (*n* = 236,568) had an ICD code for only one type of strabismus, with most of the remaining patients having multiple ICD code types (mean number ICD code types = 1.36; max = 9).

It should be noted that there were patients also identified who had a CPT code specific to strabismus surgery but who had no ICD code for strabismus present that could be identified from the EHR tables used (*n* = 1532).

### 3.3. Strabismus Surgery

A total of 7399 VHA patients (2.30% of those with a strabismus diagnosis) had at least one strabismus surgery documented in the EHR between 2000 and 2022, with a total of 9444 strabismus surgeries identified. A majority had one strabismus surgery, whereas 1373 (18.55%) underwent multiple surgeries (1–10 surgeries, mean = 1.28). The CPT code for strabismus surgery on one horizontal muscle in one eye (67311) was the most frequently used surgical code, followed by codes for two horizontal muscles in one eye (67312), and one vertical muscle (67314) (Table 2). The CPT code 67332, which is used to indicate reoperations or operations on muscles with scarring, was used in 953 surgeries. A total of 2885 surgeries had more than one CPT code (1–8 CPT codes, mean = 1.44). Although intended as add-on codes only, codes for muscle transpositions, prior non-strabismus eye surgery, prior strabismus surgery or restrictive myopathy, and placement of either posterior fixation or adjustable sutures (67320, 67331, 67332, 67334, and 67335, respectively) were identified as the only code in 1616 surgeries. Four thousand five hundred and eighty-seven surgeries (48.57%) had an associated ICD code for strabismus documented in the EHR, with the most common code type being for “exotropia” (*n* = 3659), followed by “esotropia” (*n* = 1659) and “other and unspecified heterotropia including vertical” (*n* = 1368) (Table 3).

### 3.4. Demographic Associations

Consistent with reported demographics of VHA-enrolled Veterans, a majority of these strabismus cases were male (93.6%), white (74.9%), non-Hispanic (87.5%), and older (64% ≥60 years of age) (Table 4) [27]. Two-thirds of patients with strabismus also had a history of a medical condition determined to be connected to their military service. When comparing the demographics of patients with a strabismus diagnosis who either did or did not undergo strabismus surgery, there was a significant difference in racial, ethnic, and age composition (Table 4). Among patients with strabismus who underwent surgery, a higher proportion were female, African American, non-Hispanic, or in the age groups 29 years and younger or between 40 and 69 years.

Since annual enrollment by sex, race, ethnicity, and age group is available through the VA, annual prevalence estimates and incidence rates stratified by demographic were also calculated (Table 5). Strabismus prevalence differed significantly by sex, race, ethnicity, and age; prevalence was highest among patients that were: male, white, non-Hispanic, and older, with the highest prevalence among those aged 60–69 and 70–79 years. Strabismus incidence rates also differed significantly by race, ethnicity, and age, with the highest rates in white and non-Hispanic patients, as well as patients 60–69 years.

**Figure 4:**
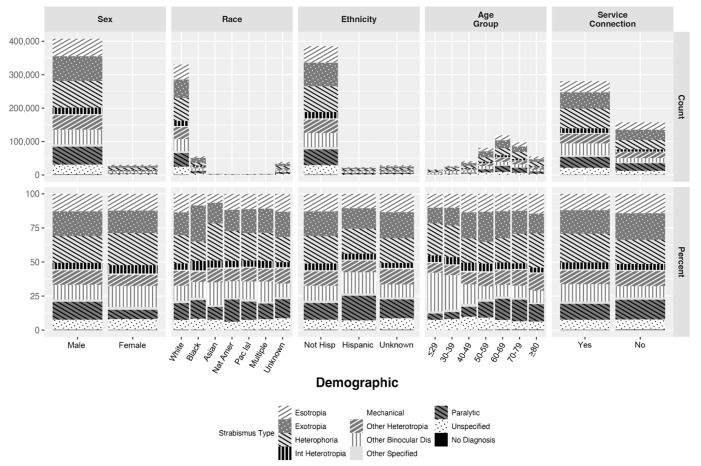
Distribution of strabismus diagnosis types by demographic. Absolute counts of each strabismus diagnosis type by sex, race, ethnicity, age group, and a history of a service-connected disability are shown in the top row of box plots. The bottom row shows percentage of each diagnosis type of total diagnoses by each demographic to better visualize the distribution of strabismus types in demographics with smaller absolute population. Strabismus types (key below plots) and demographic categories (x-axis) correspond to Table 3 and Table 4, respectively. Abbreviations: Nat Am = Native American or Alaskan Native, Pac Isl = Native Hawaiian or other Pacific Islander; Int heterotropia = Intermittent heterotropia; Other Binocular Dis = Other disorders of binocular eye movement.

There were significant relationships between the distribution of strabismus types and age group, sex, race, ethnicity, and a history of service-connected disability (*p* < 0.001 for all demographics). The differences in strabismus types by sex remained significant after adjusting for age, and the differences by race, ethnicity, and history of service connection remained significant after adjusting for age and sex. The number of patients with each strabismus type for each demographic, as well as the percent of diagnoses for each strabismus type within these demographics, are shown in Figure 4.

## 4. Discussion

Real-word data from electronic health records are increasingly being utilized as a tool to inform health and disease identification and treatment strategies and serve as a complement or alternative to standard clinical trials [36]. The VHA EHR and CDW have the potential to provide a wealth of information on a sizable patient population representing approximately half of military Veterans in the U.S. The research tools available through VINCI allow a better understanding of the frequency, risk factors, and treatment of diseases in this population, with the ultimate goal of implementing policies to improve access to and quality of VHA care. Despite an increased demand for eye services among Veterans, studies to characterize eye disease at the national level in the VHA have been limited [37]. Two national-level reports have examined cataract surgery complications in Veterans receiving surgery at the VHA versus those who did not [14,16]. Wu et al., compared cohorts of 1.8 million VHA patients and 1.2 million Medicare patients and found that a higher proportion of Medicare patients received cataract surgery [18]. Finally, a small number of other reports have used national CDW data accessed through VINCI to address questions of eye disease risk and eye care delivery, including resident involvement in cataract surgery from 2010–2021, the relationship between age-related macular degeneration diagnosis and COVID-19 outcomes, the presence of ocular inflammation in COVID-19 patients, and a comparison of driving distance for Veterans receiving cataract surgery at a VHA facility versus in the community [12,13,15,17].

By utilizing data from the VHA EHR from its implementation in 2000 through 2022, this study adds to our understanding of the prevalence of strabismus in American adults, specifically Veterans. The peak prevalence estimate reported here, 2.29% in FY2022, falls well within the range of other studies of adult strabismus; population-based studies of heterotropia based upon ocular examination range from 1.1% in Danish adults to an estimated 5–6% of American adults in the 1971–1972 NHANES [2,3,4,5,6,7]. The single-year point prevalence estimate using ICD codes in a 5% sample of the Medicare population was lower at 0.68%, in part due to methodologic differences. In the Medicare population, prevalence was calculated using only the number of ICD code diagnoses reported in a given year per population [8]. In this study, diagnoses were cumulative, with any non-deceased patient with a prior ICD code for strabismus in the EHR included in subsequent years’ prevalence calculations, thus increasing the calculated prevalence in the VHA over time. This trend would be expected to continue until new diagnoses and deaths of strabismus patients reach an equilibrium; as this did not occur during the study time period, it suggests that the true prevalence of strabismus in the VHA may be higher.

Strabismus incidence data from Olmsted County, Minnesota, reported by The Rochester Epidemiology Project, serve as another comparison for this study since medical record data from an equivalent time frame were utilized [10]. One major difference, however, is that the Rochester Epidemiology Project study reported only new diagnoses of adult-onset strabismus, which was confirmed by a review of medical records. As our study used ICD codes, it did not distinguish between adult-onset and childhood-onset strabismus persisting into adulthood. Furthermore, more stringent diagnostic criteria were used, such as the inclusion of heterophorias only if associated with diplopia as compared with the inclusion of all ICD codes for heterophoria. These differences result in a much higher rate of new diagnoses (168.9 per 100,000 in the VHA compared with 54.1 per 100,000 in the Olmsted County data) and a higher lifetime risk of a strabismus diagnosis in the VHA population.

While strabismus prevalence and incidence rates appear elevated over other U.S. adult populations, the rate of strabismus surgery among VHA patients diagnosed with strabismus was similar to previously reported data. A total of 2.30% of VHA patients with a strabismus diagnosis had surgery, as compared to 2.54% of Medicare patients with a diagnosis of strabismus [10]. Adults aged ≥20 years in the IRIS registry were slightly more likely to undergo surgery, with 3.54% of patients with a strabismus diagnosis undergoing surgery, an observation consistent with the nature of the IRIS registry. Since the eye care providers coding for strabismus in the IRIS registry may be more likely to be strabismus specialists than providers in the VHA, they may also be more likely to offer surgery [11].

One of the most notable findings in this study is that among patients with a strabismus diagnosis, patients undergoing surgery were more likely to be African American. This contrasts with African American patients having the lowest incidence rate of strabismus of any racial demographic in this study and the second lowest prevalence (unknown race excepting). This observation further contrasts with findings reported in the Medicare population, where African American patients were significantly less likely to undergo strabismus surgery, as well as a report on cataracts in the VHA, where African American patients were less likely than white patients to receive cataract surgery within five years of diagnosis [9,18].

Several key differences were identified when comparing the types of strabismus diagnosed in the VHA population with other populations. Although the population studies suggest that the prevalence of heterophoria may be as high as 20–30%, the higher proportion of patients with a code for heterophoria in our dataset compared with the Medicare and IRIS registry data is notable [2,5,8,11]. Potential explanations for this higher frequency include a true increased rate of larger angle or symptomatic heterophorias, over-coding of small asymptomatic heterophorias by providers without subspecialty training in strabismus, or misdiagnosis of other strabismus types (e.g., a patient with an intermittent exotropia is coded as an exophoria), the latter of which has clinical implications for Veterans who may benefit from treatment of their strabismus.

With regards to horizontal strabismus, the proportion of exotropia and esotropia diagnoses in the total VHA population is notable. The proportion of these two diagnoses in adults is known to vary by population, with Asian populations reported to have more exotropia diagnoses and European populations more likely to have esotropia diagnoses [3,4,5,6,7]. In U.S. adults, the 1971–1972 NHANES found a higher proportion of exotropia diagnoses compared with esotropia diagnoses in all those studied but with higher proportions among African American respondents compared with white respondents [2]. In contrast, in the Rochester Epidemiology Project and Medicare populations, nearly equal numbers of exotropia and esotropia diagnoses were reported [8,10]. The VHA population, which has a lower proportion of White patients and a higher proportion of African Americans than either of those two studies, had a higher number of exotropia diagnoses (XT:ET ratio = 1.40) [8,10]. This difference may, in part, be explained by racial differences. Consistent with prior literature, African American and Asian VHA patients had a higher XT:ET ratio than White VHA patients (Figure 4) [2,4,5,7]. However, when horizontal strabismus diagnoses from White patients in the same age range as Medicare patients (≥65 years and older) were examined, exotropia diagnoses were still more common than esotropia diagnoses (XT:ET ratio = 1.14). While it is possible that this is due to health system factors (e.g., providers in the VHA being more likely to identify and document exotropia than esotropia), the XT:ET ratio in the VHA may act as a marker for other medical comorbidities in the Veteran population associated with exotropia.

Demographic factors had a significant relationship with the types of strabismus diagnoses identified in the VHA. Age group had a significant effect on the distribution of strabismus types, with paralytic strabismus unsurprisingly representing a higher proportion of strabismus diagnoses in patients aged ≥50 years than patients < 50 years. Conversely, “Other disorders of binocular eye movement”, represented a higher proportion of diagnoses in Veterans aged <50 years. The latter category—represented by ICD9 codes 378.8x and ICD10 codes H51.0x–H51.8x, which includes codes for convergence insufficiency, conjugate gaze palsy, and internuclear ophthalmoplegia—was used in over 15% of Veterans with a strabismus diagnosis. A majority of the diagnoses from this ICD category in the VHA (71.08%) were for convergence insufficiency, which has been identified in a sizable percentage of VHA patients with TBI [19,20,21]. Similar to heterophoria diagnoses, patient and/or provider factors could also contribute to the number of convergence insufficiency diagnoses found in the VHA.

Comparison of the distribution of strabismus diagnoses by other demographic factors revealed several demographic differences. For instance, comparison by sex revealed a larger proportion of ICD codes for paralytic strabismus and vertical heterotropias in males compared with a higher proportion of codes for “Other disorders of binocular movement” in females. Although the mean age of males in this patient cohort is older than that of females, the relationship between strabismus type and sex remained after adjusting for age. Differences by race, although significant, were limited by small samples of most racial groups and mostly consisted of differences in the XT:ET ratio as discussed above. Comparisons by ethnicity, however, identified a higher proportion of paralytic strabismus diagnoses among patients identifying as Hispanic. Understanding this and other demographic-specific differences in strabismus type will require further investigation.

Several limitations should be considered when interpreting EHR data as presented here. First and foremost, EHR data are not primarily acquired for research purposes, but rather for clinical care, and may contain inaccuracies or inconsistencies. Perhaps most notable are the patients noted to have multiple birthdates or years of death recorded in the EHR. However, other indicators of potential data quality problems were noted, such as patients with up to nine strabismus ICD code types or surgeries with up to eight CPT code types. Fortunately, these were a very small number of patients in the context of a large study. They do, however, help to illustrate the challenge of data reliability when using electronic health records.

Interpretation of disease prevalence data based on diagnostic and procedural codes should also be performed with appropriate care. Although ICD codes were used in this study due to their ease of identification and manipulation, as well as the ability to compare with previously published data in the Medicare and IRIS registry populations, they may not accurately reflect clinical records, potentially underestimating or misrepresenting diseases [8,9,11]. Specifically, they may not fully capture disease prevalence if they are not used to bill an encounter or are not attached to a diagnosis in a patient’s problem list. This has been described in conditions with clinical complexity, such as traumatic brain injury, as well as conditions that may not be perceived as a primary diagnosis, such as obesity in hospitalized children [38,39]. In a patient with strabismus in the setting of a higher priority cerebrovascular event, for instance, their strabismus may be documented in the chart note but not included in the problem list or coded as diplopia. In CPRS, patient encounters are primarily recorded in free text notes. ICD codes may be recorded as a reason for a specific patient encounter or included in the patient problem list, but are not necessary for physician reimbursement, decreasing the incentive to meticulously document ICD codes at each encounter. Data from this study directly suggest that ICD codes for strabismus may not always be included in patients’ charts as the ICD code diagnosis was missing in 1532 patients’ charts despite having a CPT code specific to strabismus surgery. However, this discrepancy may be due to other causes, such as a strabismus CPT code being entered in error for another surgery. Prior published evidence in the VHA also suggests that EHR fields do not fully capture disease prevalence. For instance, the inclusion of natural language processing (NLP) of VHA clinical notes improved the identification of cases of pneumonia over ICD coding alone [40]. Similarly, although it did not examine ICD codes, a study comparing a screening survey in CPRS with clinical encounter records found that the survey data underestimated military sexual trauma [41]. Another effect of incomplete or inconsistent coding is that ICD codes may be included later than initial onset, complicating any study of temporal relationships. Although the time from diagnosis to surgery was of interest for this study, the data were deemed too unreliable, with a sizable number of patients having their first ICD code for strabismus documented after their first CPT code for strabismus surgery.

ICD codes may also misrepresent disease type or overestimate disease prevalence in an EHR relative to other populations if coding differs between them. For instance, the data in this study suggest that the likelihood of receiving a diagnosis of heterophoria was higher than in other studies relying on ICD codes. One possibility is that a different provider makeup in the VHA (e.g., fewer strabismus specialists and more comprehensive providers) results in increased documentation of asymptomatic heterophorias or misdiagnosis of an intermittent heterotropia as a heterophoria. Furthermore, different provider types may code the same patient in different ways. For instance, a strabismus specialist uses multiple ICD codes to describe a patient with complicated strabismus, whereas a comprehensive provider may only capture one aspect of the strabismus or simply code it as unspecified.

Incomplete or inaccurate coding in this dataset was also noted in the distribution of CPT codes. When used properly for billing, CPT codes should in theory provide a characterization of the surgery performed. Since complete CPT coding may not alter physician payment reimbursement in the VHA, the codes used may be incomplete or even incorrect, such as in the case of surgeries identified in this report with only an add-on code for strabismus surgery documented.

## 5. Conclusions

Utilizing national-level EHR data, this study identified over 300,000 Veterans in the VHA with strabismus diagnoses. Our findings suggest that VHA patients have a higher likelihood of receiving a strabismus diagnosis than comparable civilian populations in the U.S., but direct comparison between the VHA and other studies is limited by the use of billing and procedural codes in our study, which may not fully reflect true clinical diagnoses. Patient demographic characteristics were differentially associated with the prevalence of strabismus and strabismus surgery, most notable being the higher frequency of strabismus surgery in African American patients with a strabismus diagnosis. Further characterization and multivariable modeling of additional factors, including geographic region, provider type, and comorbid disease, may help to explain these differences. Ultimately, evaluation of clinical notes by either manual chart review or NLP would be desirable to more accurately characterize strabismus in the VHA population. Such investigation may identify patients with strabismus symptomatology without diagnosis codes, with implications for VHA population screening and treatment resource allocation.

## Figures and Tables

**Table 1:** Frequency of strabismus diagnoses by strabismus type among Veterans enrolled in the Veterans Health Administration, 2000–2022.

Disease Category	ICD9	ICD10	No.	Proportion of Diagnoses (%)	Proportion of Patients (%) ^a^
Esotropia	378.0x	H50.0x	56,268	12.88	17.49
Exotropia	378.1x	H50.1x	79,149	18.12	24.61
Intermittent Heterotropia	378.2x	H50.3	21,342	4.88	6.64
Other and Unspecified Heterotropia Including Vertical	378.3x	H50.2/H50.4	45,933	10.51	14.28
Unspecified Heterophoria	378.4x	H50.5	86,978	19.91	27.04
Paralytic Strabismus	378.5x	H49	55,694	12.75	17.32
Mechanical Strabismus	378.6x	H50.6	3425	0.78	1.06
Other Specified Strabismus	378.7x	H50.8	7366	1.69	2.29
Other Disorders of Binocular Eye Movement	378.8x	H51	48,771	11.16	15.16
Unspecified Eye Movement Disorder	378.9x	H51.9	31,992	7.32	9.95
No ICD, CPT only			1532		0.48

^a^ Patients may have more than one strabismus type documented and reported.

**Table 2:** Number of strabismus surgeries by associated CPT codes among Veterans enrolled in the Veterans Health Administration, 2000–2022.

CPT Code	Description	No. of Associated Surgeries ^a^
67311	1 horizontal muscle	3450
67312	2 horizontal muscles, 1 eye	2554
67314	1 vertical muscle, except superior oblique	1718
67316	2 vertical muscles, except superior oblique, 1 eye	309
67318	Any surgery, superior oblique	245
67345	Chemodenervation, any extraocular muscle	972
67320 ^b^	Transposition of extraocular muscle	351
67331 ^b^	Prior eye surgery, not involving extraocular muscle(s)	1739
67332 ^b^	Prior eye surgery on extraocular muscle(s) or restrictive myopathy	953
67334 ^b^	Posterior fixation placement	126
67335 ^b^	Adjustable suture placement	1184

^a^ Surgeries may have more than one associated CPT code. ^b^ Add-on code used in conjunction with 67311, 67312, 67314, 67316, or 67318.

**Table 3:** Frequency of surgeries by associated ICD diagnosis type among Veterans enrolled in the Veterans Health Administration, 2000–2022.

Disease Category	ICD9 Code	ICD10 Code	No. of Associated Surgeries (%) ^a^
Esotropia	378.0x	H50.0x	1659 (17.57)
Exotropia	378.1x	H50.1x	3659 (38.74)
Intermittent Heterotropia	378.2x	H50.3	437 (4.63)
Other and Unspecified Heterotropia Including Vertical	378.3x	H50.2/H50.4	1368 (14.49)
Unspecified Heterophoria	378.4x	H50.5	27 (0.29)
Paralytic Strabismus	378.5x	H49	755 (7.99)
Mechanical Strabismus	378.6x	H50.6	138 (1.46)
Other Specified Strabismus	378.7x	H50.8	74 (0.78)
Other Disorders of Binocular Eye Movement	378.8x	H51	57 (0.60)
Unspecified Eye Movement Disorder	378.9x	H51.9	442 (4.68)
Diplopia	368.2	H53.2	606 (6.42)
No Associated ICD			4737 (50.16)

^a^ Surgeries may have more than one associated strabismus type.

**Table 4:** Patient demographics for Veterans Health Administration-enrolled Veterans with a diagnosis of strabismus and for those undergoing strabismus surgery, 2000–2022.

Demographic	Total (*n* = 321,639)	Strabismus Surgery		
		No (*n* = 314,240)	Yes (*n* = 7399)	*p* ^a^
**Sex, *n* (%)**				**0.046**
Female	22,022 (6.85)	21,472 (6.83)	550 (7.43)	
Male	299,617 (93.15)	292,768 (93.17)	6849 (92.57)	
**Race, *n* (%)**				**<0.001**
American Indian or Alaska Native	2206 (0.69)	2163 (0.69)	43 (0.58)	
Asian	2730 (0.85)	2695 (0.86)	35 (0.47)	
Black or African American	40,822 (12.69)	39,540 (12.58)	1282 (17.33)	
Multiple Races	2720 (0.85)	2659 (0.85)	61 (0.82)	
Native Hawaiian or Other Pacific Islander	2327 (0.72)	2276 (0.72)	51 (0.69)	
Unknown/Declined	29,724 (9.24)	29,226 (9.30)	498 (6.73)	
White	241,110 (74.96)	235,681 (75.00)	5429 (73.37)	
**Ethnicity, *n* (%)**				**<0.001**
Hispanic or Latino	17,490 (5.44)	17,146 (5.46)	344 (4.65)	
Not Hispanic or Latino	281,406 (87.49)	274,743 (87.43)	6663 (90.05)	
Unknown/Declined	22,743 (7.07)	22,351 (7.11)	392 (5.30)	
**Age at Diagnosis, *n* (%)**				**<0.001**
29 and younger	12,467 (3.88)	12,155 (3.87)	312 (4.22)	
30–39	20,200 (6.28)	19,743 (6.28)	457 (6.18)	
40–49	28,263 (8.79)	27,266 (8.68)	997 (13.47)	
50–59	56,792 (17.66)	54,931 (17.48)	1861 (25.15)	
60–69	86,300 (26.83)	84,200 (26.79)	2100 (28.38)	
70–79	74,168 (23.06)	72,888 (23.20)	1280 (17.30)	
80 and older	43,449 (13.51)	43,057 (13.70)	392 (5.30)	
**Any Service-Connected Condition, *n* (%)**				**0.616**
N	118,984 (36.99)	116,268 (37.00)	2716 (36.71)	
Y	202,655 (63.01)	197,972 (63.00)	4683 (63.29)	

^a^ Strabismus surgery versus no strabismus surgery by demographic.

**Table 5:** Prevalence and Incidence Data and Statistical Comparisons by Demographic for Veterans Enrolled in the Veterans Health Administration during Fiscal Years 2002–2022 (1 October 2002 to 30 September 2022).

Demographic Factor	Annual Prevalence (%)	*p*	Incidence Rate (per 100,000)	*p*
	FY22 (Range)		Median (Range)	
**Sex**		**<0.001**		**0.1**
Male	2.31 (0.41–2.31)		171.4 (131.9–199.8)	
Female	2.13 (0.44–2.13)		170.8 (123.5–198.5)	
**Race**		**<0.0001**		**<0.0001**
White	2.66 (2.01–2.66)		226.5 (151.0–248.5)	
Black or African American	2.15 (1.60–2.15)		181.8 (127.8–205.9)	
Asian	1.87 (1.36–1.87)		199.9 (133.9–233.0)	
Native Hawaiian or Other Pacific Islander	2.21 (1.71–2.21)		200.8 (117.7–249.9)	
American Indian or Alaskan Native	2.39 (1.85–2.39)		206.0 (129.0–305.8)	
Multiple Races	2.29 (1.34–2.36)		194.4 (145.9–266.6)	
Unknown/Declined	0.95(0.41–0.95)		58.6 (38.7–83.8)	
**Ethnicity**		**<0.0001**		**<0.0001**
Not Hispanic or Latino	2.68 (0.75–2.63)		265.9 (146.0–311.7)	
Hispanic or Latino	2.36 (0.72–2.89)		251.6 (138.0–323.9)	
Unknown/Declined	0.59 (0.16–0.70)		22.9 (18.3–55.1)	
**Age Group**		**<0.0001**		**<0.0001**
29 and Younger	0.69 (0.20–1.46)		179.1 (81.2–381.5)	
30–39	1.45 (0.23–1.45)		127.2 (78.2–155.5)	
40–49	1.54 (0.38–1.54)		139.0 (95.7–159.7)	
50–59	1.81 (0.45–1.81)		182.2 (109.1–205.3)	
60–69	2.63 (0.47–2.64)		199.8 (152.1–261.8)	
70–79	3.03 (0.43–3.03)		176.9 (143.4–228.8)	
80 and Older	2.64 (0.36–2.64)		140.8 (118.2–188.6)	

## Data Availability

The datasets presented in this article are not readily available because they are confidential patient data from the VHA healthcare system. Access to the patient data used is restricted to approved VHA researchers and is subject to VHA rules and regulations. Information on VINCI and CDW data may be found at the VA Office of Research and Development website (https://research.va.gov).
